# Effects of Extreme Weight Loss on Cardiometabolic Health in Children With Metabolic Syndrome: A Metabolomic Study

**DOI:** 10.3389/fphys.2021.731762

**Published:** 2021-09-24

**Authors:** Jingxin Liu, Lin Zhu, Jing Liao, Xiaoguang Liu

**Affiliations:** ^1^School of Sport and Health, Guangzhou Sport University, Guangzhou, China; ^2^Guangdong Provincial Key Laboratory of Physical Activity and Health Promotion, Guangzhou Sport University, Guangzhou, China

**Keywords:** children with obesity, metabolic syndrome, metabolomics, exercise, cardiometabolic health

## Abstract

**Objectives:** To evaluate the effect of extreme weight loss programs on circulating metabolites and their relationship with cardiometabolic health in children with metabolic syndrome.

**Methods:** This study was a quasi-experimental design with a pretest and post-test. Thirty children with metabolic syndrome and aged 10–17years were recruited to an extreme weight loss program (i.e., exercise combined with diet control). The primary outcomes included plasma metabolites, body composition, and cardiometabolic risk factors. A total of 324 metabolites were quantitatively detected by an ultra-performance liquid chromatography coupled to tandem mass spectrometry system, and the variable importance in the projection (VIP) value of each metabolite was calculated by the orthogonal projection to latent structures discriminant analysis. The fold change (FC) and *p* value of each metabolite were used to screen differential metabolites with the following values: VIP>1, *p* value<0.05, and |log2FC|>0.25. Pathway enrichment and correlation analyses between metabolites and cardiometabolic risk factors were also performed.

**Result:** A large effect size was observed, presenting a weight loss of −8.9kg (Cohen’s *d*=1.00, *p*<0.001), body mass index reduction of −3.3kg/m^2^ (Cohen’s *d*=1.47, *p*<0.001), and body fat percent reduction of −4.1 (%) (Cohen’s *d*=1.22, *p*<0.001) after the intervention. Similar improvements were found in total cholesterol (Cohen’s *d*=2.65, *p*<0.001), triglycerides (Cohen’s *d*=2.59, *p*<0.001), low-density lipoprotein cholesterol (Cohen’s *d*=2.81, *p*<0.001), glucose metabolism, and blood pressure. A total of 59 metabolites were changed after the intervention (e.g., aminoacyl-tRNA biosynthesis, glycine, serine, and threonine metabolism; nitrogen metabolism, tricarboxylic acid cycle, and phenylalanine, tyrosine, and tryptophan biosynthesis). The changes in metabolites (e.g., amino acids, fatty acids, organic acids, and carnitine) were related to lipid metabolism improvement (*p*<0.05). Organic acids and carnitines were associated with changes in the body composition (*p*<0.05).

**Conclusion:** Exercise combined with dietary control improved the body composition and cardiometabolic health in children with metabolic syndrome, and these changes may be related to plasma metabolites.

## Introduction

Childhood obesity is closely related to the clustering of cardiometabolic risk factors, such as dyslipidemia, hypertension, and insulin resistance, which contribute to the development of metabolic syndrome and an increased risk of developing co-morbidities, including type 2 diabetes and cardiovascular disease in adulthood (Juonala et al., 2011; Buscot et al., 2018). Weight loss is an effective strategy for reducing cardiometabolic risk in children with metabolic syndrome. Physical activity, which has attracted increasing amount of attention, can not only effectively reduce weight but also improve cardiometabolic health and increase cardiovascular fitness (Dias et al., 2018; Hansen et al., 2018; Labayen et al., 2020; Fridolfsson et al., 2021). A randomized controlled trial by Davis et al. also showed that moderate-intensity aerobic exercise (heart rate approximately 150 beats/min) reduced body composition and the risk of diabetes effectively and improved glucose metabolism in overweight and obese children (Davis et al., 2012). Moreover, a recent meta-analysis showed that high-intensity and moderate-intensity exercises can reduce body weight and improve cardiometabolic health in obese children (Liu et al., 2020). These findings suggest that moderate- or high-intensity exercises benefit the reduction of weight and promotion of cardiometabolic health in obese children. Furthermore, emerging evidence has shown that extreme weight loss programs that combine exercise with diet control can lead to a great weight loss in a short period of time. Hutchesson et al. discovered that an 8-week Biggest Loser Club with −500kcal/day less than the estimated energy expenditure resulted from an exercise combined with diet intervention can reduce weight by −4.5kg (Hutchesson et al., 2013). Kerns et al. (2017) observed that diet restriction and vigorous physical activity intervention in the Biggest Loser Competition can achieve rapid weight change. However, the effects of extreme weight loss programs on cardiometabolic health have been inconsistent between studies (Liu et al., 2015; Xu et al., 2018; Yang et al., 2018), and most of the research to date has focused on the effects of extreme weight loss programs on weight loss and cardiometabolic health; furthermore, discussion on the underlying mechanisms is still lacking.

Metabolomics is defined as an “omics” technology characterized by the high-throughput identification and quantification of small-molecule (<1,500Da) metabolites in a cell, tissue, blood, or organism (Nicholson et al., 1999; Johnson et al., 2016). Plasma metabolites were early diagnostic markers for obesity-related type 2 diabetes and cardiovascular disease (Meyer et al., 2018; Short et al., 2019; Perng et al., 2020), plasma branched-chain amino acids (BCAAs), aromatic amino acids (AAAs), acylcarnitine, and incomplete oxidized lipid metabolites, which are associated with metabolic abnormalities in children with obesity (Newgard, 2012; Wahl et al., 2012; McCormack et al., 2013; Zhou et al., 2019). However, whether extreme weight loss programs can cause changes in small-molecule metabolites, and whether changes in small-molecule metabolites are associated with weight loss and cardiometabolic health improvement in children with metabolic syndrome remain unclear. Thus, this study aimed to evaluate the effects of extreme weight loss programs on circulating metabolites and their relationship with cardiometabolic health in children with metabolic syndrome.

## Materials and Methods

### Study Design and Participants

From June 2019 to August 2019, 30 children with metabolic syndrome were screened and recruited from 103 obese children aged 10–17years who participated in the Biggest Loser Training Camp (Shenzhen, China). Metabolic syndrome was defined under the definition and prevention and treatment of metabolic syndrome in Chinese children and adolescents (Liang Li and Fu Junfen, 2012); children with central obesity were defined to have a waist circumference (WC) higher than the 90th percentile for age and sex, and they should meet any two of the following criteria: fasting plasma glucose (FPG)≥5.6mmol/L; high-density lipoprotein-cholesterol (HDL-c)<1.03mmol/L or non-HDL-c≥3.76mmol/L; hypertension defined as systolic blood pressure (SBP) or diastolic blood pressure (DBP) in the 95th percentile or higher for age and sex; triglyceride (TG)≥1.47mmol/L. All children and their parents were notified of the benefits and potential risks in this study before the intervention. A written informed consent was obtained from all children and their parents, and the study protocol was approved by the Ethical Committee of the Guangzhou Sport University (No. 2018LCLL-008).

The present study was a quasi-experimental design with pretest and post-test. The participants performed a standardized exercise combined with diet control under an extreme weight loss intervention for 30days. To ensure then-effective implementation of the intervention, experienced coaches and researchers were tasked to manage the participants and monitor and record the exercise and diet intake. Anthropometric data, body composition, and cardiometabolic risk factors (i.e., blood glucose, blood lipid, and blood pressure) were measured at pre- and post-intervention. The pretest was performed before the beginning of the extreme weight loss intervention, whereas the post-test was conducted 12h after the last exercise training.

### Standardized Exercise Combined With Diet Control

#### Diet Control

The participants were instructed to follow a regular diet habit, with breakfast from 8:00 to 8:30, lunch from 11:30 to 12:00, and dinner from 17:30 to 18:00. The diet control of each participant was designed based on the resting energy expenditure (REE). The REE was measured for each participant before the intervention using indirect calorimetry methods. The concentrations of O_2_ and CO_2_ were measured by a gas metabolizer (Cortex Meta Max 3B, Germany) for 15min. Weir’s equation was used to calculate the REE and resting metabolic rate (RMR): $REE\;\left(kcal/\min\right)=3.9*VO_{2}\left(L/\min\right)+1.1*VCO_{2}\left(L/\min\right)$; $RMR\left(kcal/day\right)=REE*1440$. To match the dietary intake with RMR, nutrition experts designed the diet in accordance with the Chinese Food Composition Table compiled by the Chinese Center for Disease and Prevention. The types of food included fruits, vegetables, grains, legumes, eggs, meat, and dairy products. The ratios of energy intake for breakfast, lunch, and dinner were 30, 40, and 30%, respectively.

#### Exercise Intervention

The exercise intervention was the main part of the extreme weight loss intervention, and it lasted for 240min per day from 09:30 to 11:30am and 15:30 to 17:30pm. To ensure that all participants performed the exercise intervention effectively, we confirmed that the design of the exercise intervention program followed the principles of individuation, gradualism, interest, and safety. Considering the results of current studies mentioning that moderate- or high-intensity exercise can effectively improve the cardiometabolic health of obese children, the exercise intensity of the extreme weight loss intervention was mainly that of a moderate-intensity aerobic exercise combined with a short period of high-intensity exercise. The exercise types were mainly outdoor hiking, fast walking, jogging, sports games, aerobic exercises, recreational ball games, etc. The exercise intensity was monitored by heart rate, and the participant’s heart rate during exercise was kept in the range of 50–80% HR_max_. During the exercise intervention, researchers observed the participants’ response to the exercise intervention and recorded and adjusted the exercise intensity based on the conditions of each participant. Each training session started with a 30-min warm-up, followed by an 80-min training session, and ended with a 10-min cooldown session. All exercise sessions were supervised by a qualified conditioning coach. Supplementary Table S1 shows the detail of diet control.

### Data Collection and Procedure

#### Anthropometric Measurements and Body Composition

Anthropometric measurements, including weight, height, WC, hip circumference (HC), waist-to-hip ratio (WHR), and waist-to-height ratio (WHtR), were performed for all children at pretest and post-test. Height was measured to the nearest 0.1cm using a standard height meter, and weight was measured to the nearest 0.1kg on a digital scale. Body mass index (BMI) was calculated by weight in kilograms divided by the square height in meters. Waist circumference was measured to the nearest 0.1cm using a plastic tape while maintaining the measuring tape level. WHR was calculated by WC (cm) divided by HC (cm), and WHtR was calculated as WC (cm) divided by height (cm). Whole-body composition measurements, including fat-free mass (FFM), fat mass (FM), skeletal muscle mass (SMM), and body fat percentage (BFP), were measured using a body composition analyzer (T-SCAN PLUS, Korea). Anthropometric measurements and body composition assessment were performed by an expert with 2years of background experience following the standard measurement methods. The body composition measurements were obtained in the morning (08:00–09:00am) without eating.

#### Cardiometabolic Risk Factor Measurement

Blood pressure was measured thrice by an electronic blood pressure monitor (OMRON HEM-1020, China) in the morning after sitting for 10–15min. The mean of the closest two tests was used to record the SBP and DBP. Mean arterial pressure (MAP) was calculated from the SBP and DBP with the following formula: MAP=DBP+(SBP-DBP)/3. With heparin sodium as an anticoagulant, the fasting plasma samples were acquired *via* the antecubital vein at baseline and again at 12h after the last intervention at 30days. After standing for 30min, the plasma was separated by centrifugation at 4°C (10min at 1000g), frozen in liquid nitrogen, and stored at −80°C. The concentrations of HDL-c, low-density lipoprotein cholesterol (LDL-c), TG, and total cholesterol (TC) were measured by enzymatic assay. The FPG was measured using the glucose oxidase method. Fasting insulin (FIN) was measured using the enzyme-linked immunosorbent assay. Homeostatic model assessment for insulin resistance (HOMA-IR) was performed using the following formula.

$$HOMA-IR=\frac{FINs\left(µU/L\right)*FPG\left(mmol/L\right)}{22.5}$$

#### Metabolomic Analysis

An ultra-performance liquid chromatography coupled to tandem mass spectrometry (UPLC-MS/MS) system (ACQUITY UPLC-Xevo TQ-S, Waters Corp., Milford, MA, United States) was used to quantitatively determine 324 metabolites, including carbohydrates, amino acids, fatty acids, organic acids, and bile acids (Xie et al., 2021).

The standard compounds of 324 metabolites and stable isotope-labeled internal standards were obtained from Sigma-Aldrich (St. Louis, MO, United States), Steraloids Inc. (Newport, RI, United States) and TRC Chemicals (Toronto, ON, Canada). Supplementary Table S2 shows the details of all metabolites. Methanol (Optima LC-MS), acetonitrile (Optima LC-MS), and isopropanol (Optima LC-MS) were commercially purchased from Thermo-Fisher Scientific (Fairlawn, NJ, United States). Formic acid was analytically pure and obtained from Sigma-Aldrich (St. Louis, Mo, United States). The ultrapure water was produced by a Mill-Q reference system equipped with a LC-MS Pak filter (Millipore, Billerica, MA, United States). All standard components were weighed and dissolved in water, methanol, sodium hydroxide solution, or hydrochloric acid solution to obtain a single, standard component reserve solution with a concentration of 5.0mg/mL. An appropriate amount of each standard component reserve solution was used to prepare a mixed standard component reserve solution.

To diminish sample degradation, we thawed the plasma sample on an ice bath and added 25μl of it to a 96-well plate. Then, 100μl ice-cold methanol with a partial internal standard was automatically added to each sample at Biomek 4,000 workstation (Biomek 4,000, Beckman Coulter, Inc., Brea, California, United States) and mixed for 5min after intense vortexing. Next, the samples were centrifuged for 30min at 4000g/min (Allegra X-15R, Beckman Coulter, Inc., Indianapolis, IN, United States). A total of 30μl supernatant was transferred to a clean 96-well plate, and 20μl freshly prepared derivative reagents were added to each well in the workstation. The 96-well plate was sealed and followed by derivatization, which was carried out at 30°C for 60min. After derivatization, the sample was diluted with ice-cold 50% methanol solution in 350μl. The plates were placed at −20°C for 20min and then centrifuged at 4°C (4,000g, 30min). The supernatant (135μl) was transferred to a new 96-well plate with 15μl internal standard to each well. Finally, the serial dilutions of derivatized stock standards were added to the left of the 96-well plate and sealed for analysis.

ACQUITY UPLC BEH C18 1.7μm VanGuard pre-column (2.1×5mm^2^) and ACQUITY UPLC BEH C18 1.7μm analytical column (2.1×100mm^2^) were used for separation with the column temperature set at 40°C and the sample manager temperature at 10°C. The mobile phases were water with 0.1% formic acid (A) and acetonitrile/IPA (90:10, B). The initial gradient was 5% B and kept for 1min, increased to 80% B at 12min, increased to 95% B at 15min, increased to 100% B at 16min, kept at 100% B until 18min, switched back to the initial condition at 18.1min, and held until 20min. The flow rate was 0.40ml/min, and the injection volume was 5.0μl. The capillary voltages were 1.5 (ESI+) and 2.0 Kv (ESI−), and the source temperature was set at 150°C. The desolvation temperature was set at 550°C with a desolvation gas flow rate of 1,000L nitrogen per hour.

QuanMET software (v2.0, Metabo-Profile, Shanghai, China), which can perform peak integration, calibration, and quantitation for each metabolite, was used to process the raw data generated by UPLC-MS/MS.

### Quantification and Statistical Analysis

The Shapiro–Wilk and Kolmogorov–Smirnov tests were used to determine the normality of data distribution. The continuous variables were reported as means ± standard deviation (*SD*) with normal distribution, whereas the median and interquartile range (IQR) were applied to denote the non-normally distributed data. The baseline characteristics between boys and girls were compared with an independent sample t test for normally distributed continuous variables. Paired sample t test and Mann–Whitney test were used for comparison before and after intervention depending on data normality. For each outcome, the effect size (Cohen’s *d*) was calculated as $\mathrm{Cohen}’s\;d=\left(pre\_test-\mathrm{post}\_\mathrm{test}\right)/pooled\;SD$ and defined as trivial (<0.2), small (≥ 0.2, < 0.5), moderate (≥ 0.5, < 0.8), and large (≥ 0.8). Statistical analysis was performed with SPSS version 20.0 (SPSS, Inc., Chicago, IL, United States), and the statistical significance level was set at 0.05.

Multivariate statistical analyses, including principal component analysis (PCA), orthogonal projection to latent structures discriminant analysis (OPLS-DA), and univariate statistical analyses including t test and Mann–Whitney Wilcoxon test (U test), were performed to obtain the differential metabolites. First, PCA was conducted to examine the cluster of samples and identify outliers before and after the intervention. Second, OPLS-DA was performed to visualize the changes between the baseline and post-intervention. A seven-round cross-validation was carried out to validate the model against over-fitting of the OPLS-DA models, and Q2Y, R2X, and R2Y were used to quantify the interpretation of models. Q2Y suggests the model’s predictive accuracy, whereas R2X and R2Y represent the fraction of the variance of the X and Y matrixes, respectively. Cumulative values of R2X, R2Y, and Q2Y close to 1.0 indicate an excellent model with a reliable predictive capability. The variable importance in the projection (VIP) value of each metabolite was used as the criterion for metabolite screening. The fold change (FC) was displayed, and the *p* value of each metabolite was used to screen differential metabolites. To reduce the error rate, the *p* value of each differential metabolite was adjusted by a false discovery rate (FDR) method in pretest and post-test comparisons. The selection of differential metabolites was based on the following criteria: VIP>1, p value <0.05, and |log2FC|>0.25.

Metabolic pathway analysis was performed for differential metabolites to determine which metabolic pathway changed after the intervention, and the metabolic pathway analysis used the HSA sets by Kyoto Encyclopedia of Genes and Genomes (KEGG). Pathway impact was derived from the centrality normalization of the differential metabolite nodes and their sum. The pathway impact score was used to assess the importance of differential metabolites in the metabolic pathway before and after intervention. The greater the pathway impact, the more important the differential metabolites were in the metabolic pathway. To verify whether the differential metabolites were associated with improvements in the body composition and cardiometabolic health, we further performed the Spearman correlation analysis. Statistical algorithms were adapted from the widely used statistical analysis software packages in R studio.^1^

## Results

### General Characteristics and Changes in Cardiometabolic Risk Factors After Intervention

A total of 103 obese children were recruited from the Biggest Loser Train Camp program, and 30 children, including 18 boys and 12 girls, were selected for metabolic syndrome (Table 1). A large effect size was observed in the body composition following intervention, including a weight loss of −8.9±3.42kg (Cohen’s *d*=1.00, *p*<0.001), BMI reduction of −3.3±1.16kg/m^2^ (Cohen’s *d*=1.47, p<0.001), FM reduction of −6.1±2.7kg (Cohen’s *d*=1.33, *p*<0.001), and BFP reduction of −4.1%±2.1% (Cohen’s *d*=1.22, *p*<0.001) after the intervention (Table 2). Our results also revealed a decrease in body circumference, including *a*−8.6±4.1cm (Cohen’s *d*=1.39, *p*<0.001) reduction in WC and *a*−7.3±3.4cm (Cohen’s *d*=1.32, *p*<0.001) reduction in HC (Table 2). Our results also showed an improvement in cardiometabolic health and body composition after the intervention. In terms of lipid metabolism, our results showed that TC, TG, and LDL-c decreased by −1.14±0.75 (Cohen’s *d*=2.65, *p*<0.001), −1.20±0.64 (Cohen’s *d*=2.59, *p*<0.001), and−0.97±0.60mmol/L (Cohen’s *d*=2.81, *p*<0.001), respectively. Similar to the improvement of lipid metabolism, glucose metabolism, and blood pressure improved after the intervention, whereas FPG, FINs, HOMA-IR, SBP, DBP, and MAP decreased (Table 2).

**Table 1:** Baseline participant characteristics.

Characteristics	Boys (*n* =18)	Girls (*n* =12)	Total (*n* =30)	Value of *p*
Age (years)	12.6 ± 1.9	13.3 ± 1.5	12.9 ± 1.8	0.318
Height (cm)	167.4 ± 10.7	158.4 ± 8.2	163.8 ± 10.6	0.020
Weight (kg)	87.7 ± 18.0	80.5 ± 12.3	84.8 ± 16.1	0.239
Body mass index (BMI; kg/m^2^)	31.0 ± 4.0	32.02 ± 4.1	31.4 ± 4.0	0.502
RMR (kcal/day)	2445.23 ± 468.35	2129.51 ± 427.51	2318.97 ± 471.86	0.072

*Data are expressed as means±SD; independent sample t test was used for comparison*.

**Table 2:** Changes in clinical characteristics and cardiometabolic risk factors in children with metabolic syndrome.

Outcomes	Pre-intervention	Post-intervention	Changes	Cohen’s *d*	Value of *p*
Weight (kg)	84.8 ± 16.1	75.9 ± 14.4	−8.9 ± 3.4	1.00	1.10E-14
BMI (kg/m^2^)	31.4 ± 4.0	28.1 ± 3.8	−3.3 ± 1.2	1.47	1.36E-15
FM (kg)	28.8 ± 8.2	22.7 ± 7.4	−6.1 ± 2.7	1.33	3.41E-13
Fat-free mass (kg)	56.0 ± 10.0	53.2 ± 9.4	−2.9 ± 1.5	0.51	2.66E-11
Skeletal muscle mass (kg)	51.2 ± 9.2	48.8 ± 8.6	−2.4 ± 1.4	0.46	2.14E-10
Body fat percentage (%)	33.6 ± 5.4	29.5 ± 6.1	−4.1 ± 2.1	>1.22	7.82E-12
WC (cm)	104.9 ± 10.7	96.3 ± 10.4	−8.6 ± 4.14	1.41	3.19E-12
HC (cm)	109.0 ± 9.7	101.7 ± 9.0	−7.3 ± 3.4	>1.34	1.33E-12
WHR	0.96 ± 0.08	0.95 ± 0.09	−0.01 ± 0.02	0.20	0.001
WHtR	0.64 ± 0.08	0.59 ± 0.07	−0.05 ± 0.3	1.13	1.08E-11
TC (mmol/L)	4.52 ± 0.78	3.38 ± 0.64	−1.14 ± 0.75	2.65	3.25E-09
TG (mmol/L)	1.91 ± 0.65	0.71 ± 0.25	−1.20 ± 0.64	2.59	3.27E-11
High-density lipoprotein-cholesterol (HDL-c; mmol/L)	1.01 ± 0.21	1.08 ± 0.21	0.07 ± 0.19	0.58	8.60E-02
Low-density lipoprotein cholesterol (mmol/L)	2.75 ± 0.62	1.78 ± 0.56	−0.97 ± 0.60	2.81	8.48E-10
non-HDL-c (mmol/L)	3.51 ± 0.69	2.30 ± 0.56	−1.21 ± 0.64	3.17	2.94E-11
fasting plasma glucose (mmol/L)	5.86 ± 1.01	5.15 ± 0.75	−0.71 ± 1.18	1.26	0.003
FINs	10.80 (5.02)	8.97 (7.75)	−2.26 (6.99)	-	0.006
HOMA-IR	2.69 (1.70)	2.00 (1.99)	−0.58 (1.71)	-	0.002
SBP (mmHg)	113 ± 10	105 ± 10	−7 ± 9	1.39	9.70E-05
DBP (mmHg)	71 ± 8	64 ± 11	−7 ± 6	1.13	4.19E-07
MAP (mmHg)	85 ± 8	78 ± 10	−7 ± 5	1.27	1.54E-08

*Continuous variables were reported as means±(SD) with normal distribution, and the paired sample t test was used to test the differences. FINs and HOMA-IR were reported as median (IQR), and Mann–Whitney test was used to test the differences*.

### Selection and Identification of Discriminatory Metabolites Related to Metabolic Improvement

**Figure 1:**
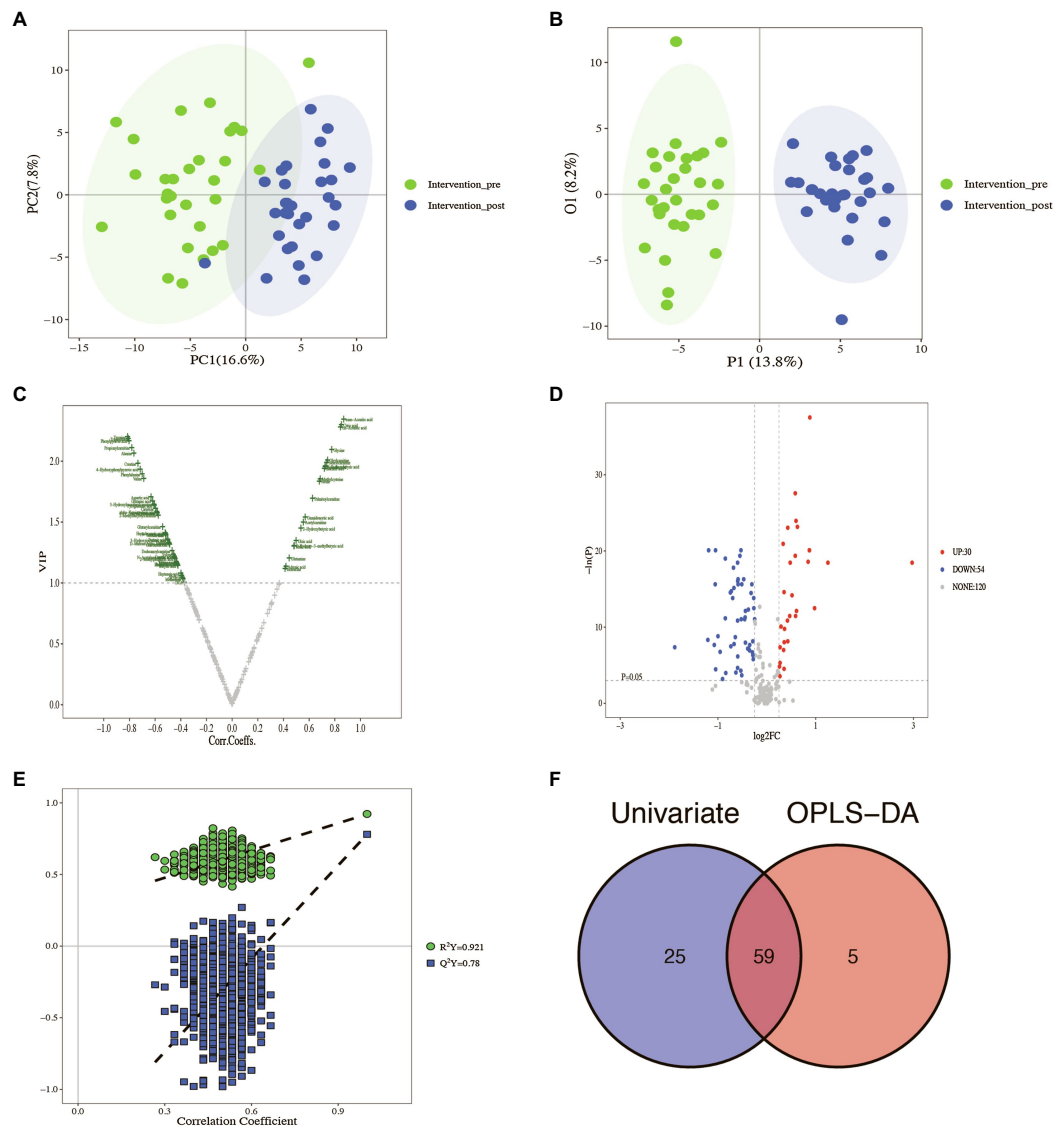
Identification of the potential metabolites between baseline and post-intervention. **(A)** PCA plot; **(B)** OPLS-DA plot; **(C)** volcano plot of OPLS-DA model; **(D)** volcano plot of univariate statistics; **(E)** OPLS-DA permutation plot; **(F)** Venn plot of differential metabolites.

All plasma samples were processed and analyzed through UPLC-MS/MS following the standardized protocol, and 204 metabolites were successfully determined in each sample. The relative abundance of each metabolite class (Supplementary Figure S1) and the separation of metabolites were evident from the PCA and OPLS-DA results (Figures 1A,B).

The scoring plot generated from a cross-validated OPLS-DA model using one predictive component and three orthogonal components further showed a distinct separation (R2Y=0.921, Q2Y=0.78; Figure 1E), indicating that the OPLS-DA model was stable and effective for fitness and prediction. Compared with the pre-intervention, the volcano plots showed that 64 metabolites were screened based on OPLS-DA with a VIP>1 (Figure 1C). The *t* test or Mann–Whitney test with the *p* value<0.05 and |log2FC|>0.25 was also carried out to validate the differential metabolites. The volcano plots showed that 30 metabolites increased, and 54 metabolites decreased after the intervention (Figure 1D). With the VIP>1, *p* value<0.05, and |log2FC|>0.25 as screening criteria, 59 differential metabolites were screened (Figure 1F, Table 3). The p value of the metabolite in Table 3 was adjusted by FDR methods, and the results showed that the pFDR of each metabolite was less than 0.05, indicating that the screened differential metabolites were more reliable (Table 3).

**Table 3:** Changes in potential metabolites in children with metabolic syndrome following the intervention.

Class	Metabolite	Value of *p*	pFDR	FC	log2FC
Amino Acids	Alanine	9.31E-09	1.01E-07	0.6596	−0.6004
Amino Acids	Aspartic acid	1.64E-07	1.24E-06	0.4813	−1.0549
Amino Acids	Creatine	1.86E-09	3.45E-08	0.4381	−1.1908
Amino Acids	Glutamic acid	1.64E-07	1.24E-06	0.6658	−0.5868
Amino Acids	Glycine	3.73E-09	5.85E-08	1.4961	0.5812
Amino Acids	Homocitrulline	1.82E-05	7.35E-05	0.6599	−0.5997
Amino Acids	Isoleucine	1.04E-05	4.92E-05	1.3842	0.4691
Amino Acids	Leucine	4.60E-04	1.42E-03	0.7844	−0.3504
Amino Acids	Methylcysteine	9.31E-09	1.01E-07	2.3801	1.251
Amino Acids	N-Acetylserine	3.79E-06	2.03E-05	0.8329	−0.2637
Amino Acids	Phenylalanine	1.86E-08	1.90E-07	0.6225	−0.6839
Amino Acids	Pipecolic acid	8.56E-09	1.01E-07	1.7982	0.8465
Amino Acids	Pyroglutamic acid	1.02E-07	9.09E-07	0.6617	−0.5958
Amino Acids	Serine	9.44E-11	3.85E-09	1.3458	0.4285
Amino Acids	Tryptophan	1.86E-09	3.45E-08	0.6943	−0.5265
Amino Acids	Tyrosine	3.73E-09	5.85E-08	0.6829	−0.5502
Amino Acids	Valine	5.09E-07	3.25E-06	0.8015	−0.3192
Benzenoids	Phenylpyruvic acid	5.59E-09	8.14E-08	0.5555	−0.8481
Bile Acids	DCA	6.08E-04	1.80E-03	0.2707	−1.885
Carbohydrates	Gluconolactone	9.98E-07	5.82E-06	0.8304	−0.2681
Carbohydrates	Glyceric acid	1.64E-07	1.24E-06	0.8096	−0.3047
Carbohydrates	Maltotriose	4.60E-04	1.42E-03	0.4748	−1.0746
Carbohydrates	Xylose	2.99E-03	7.34E-03	0.8239	−0.2795
Carboxylic acids	2-Methylbutyroylcarnitine	8.01E-08	7.43E-07	0.6657	−0.5869
Carnitines	Acetylcarnitine	1.01E-05	4.90E-05	1.5007	0.5856
Carnitines	Carnitine	1.64E-07	1.24E-06	0.6998	−0.515
Carnitines	Dodecanoylcarnitine	1.60E-05	6.65E-05	0.7302	−0.4537
Carnitines	Glutarylcarnitine	8.01E-08	7.43E-07	0.722	−0.4698
Carnitines	Isovalerylcarnitine	4.71E-07	3.10E-06	0.5972	−0.7437
Carnitines	Oleylcarnitine	1.01E-12	1.03E-10	1.4978	0.5828
Carnitines	Palmitoylcarnitine	7.72E-10	2.62E-08	1.258	0.3311
Carnitines	Propionylcarnitine	1.86E-09	3.45E-08	0.4825	−1.0515
Carnitines	Stearylcarnitine	8.35E-11	3.85E-09	1.5441	0.6268
Carnitines	2-Hydroxy-3-methyl butyric acid	5.14E-06	2.56E-05	1.5201	0.6042
Fatty Acids	Adrenic acid	1.84E-05	7.35E-05	1.3389	0.421
Fatty Acids	Azelaic acid	9.98E-07	5.82E-06	0.6165	−0.6979
Fatty Acids	Heptadecanoic acid	3.86E-07	2.71E-06	0.6052	−0.7244
Fatty Acids	Heptanoic acid	2.02E-03	5.15E-03	0.6635	−0.5918
Fatty Acids	Oleic acid	4.16E-05	1.57E-04	1.2232	0.2906
Fatty Acids	AMP	1.24E-05	5.76E-05	0.7345	−0.4452
Nucleotides	2-Hydroxybutyric acid	3.79E-06	2.03E-05	1.9684	0.977
Organic Acids	trans-Aconitic acid	4.81E-17	9.82E-15	1.8366	0.877
Organic Acids	3-Hydroxybutyric acid	9.31E-09	1.01E-07	7.8566	2.9739
Organic Acids	alpha-Ketoisovaleric acid	1.57E-05	6.65E-05	0.8407	−0.2503
Organic Acids	cis-Aconitic acid	1.86E-09	3.45E-08	1.8296	0.8715
Organic Acids	Citric acid	1.86E-09	3.45E-08	1.8301	0.872
Organic Acids	Guanidoacetic acid	9.42E-09	1.01E-07	1.3953	0.4806
Organic Acids	Isocitric acid	3.95E-11	2.69E-09	1.5129	0.5973
Organic Acids	Malic acid	4.40E-07	2.99E-06	1.2751	0.3506
Organic Acids	Pyruvic acid	1.60E-05	6.65E-05	0.696	−0.5229
Phenols	4-Hydroxyphenylpyruvic acid	2.55E-07	1.86E-06	0.6265	−0.6747
Primary BAs	GCDCA	1.13E-03	3.07E-03	0.5158	−0.955
SCFAs	2-Methylpentanoic acid	5.55E-04	1.69E-03	0.6013	−0.7339
SCFAs	3-Hydroxyisovaleric acid	2.83E-04	9.63E-04	0.8201	−0.2861
SCFAs	Caproic acid	1.72E-03	4.49E-03	0.8195	−0.2871
SCFAs	Propionic acid	4.42E-06	2.31E-05	0.7692	−0.3786
SCFAs	Valeric acid	5.14E-06	2.56E-05	0.7388	−0.4367
Unknown	D-Maltose/Alpha-Lactose	3.45E-04	1.14E-03	0.7389	−0.4365
Unknown	GCA_1	2.32E-04	8.15E-04	0.4336	−1.2056

### Pathway and Correlation Analyses for Cardiometabolic Health Improvement

#### Pathway Analysis

**Figure 2:**
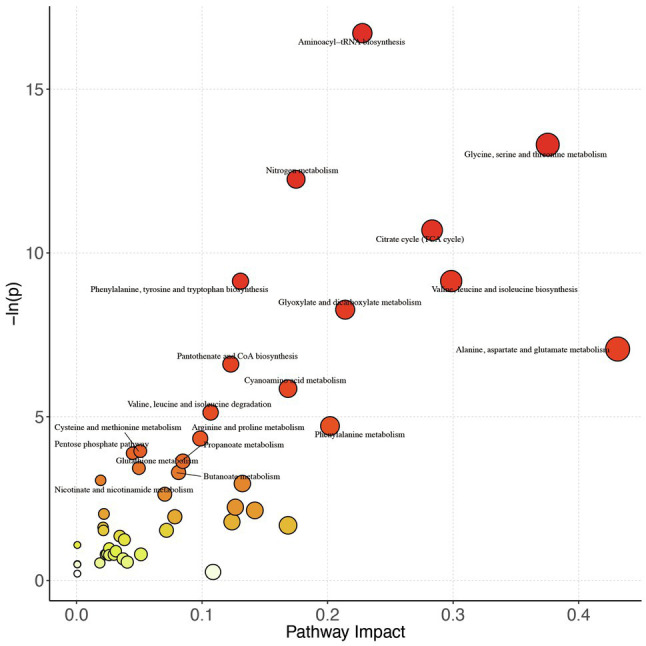
Pathway analysis bubble plot by the HSA set in KEGG. On the horizontal axis is the pathway impact, which represents the importance of differential metabolites in metabolic pathways. The vertical axis is the negative logarithm of *p* value obtained from pathway enrichment analysis. The size of pathway symbols represents the statistical significance level of pathway analysis. The color of pathway symbols represents the impact factor; large sizes and dark colors represent central pathway enrichment and high pathway impact values, respectively.

To further explore the changes in the differential metabolites after the intervention, we performed the pathway analysis by the KEGG database. A total of 48 pathways were enriched when the 59 differential metabolites were introduced into KEGG (Figure 2), and based on ln(*p* value) and pathway impact scores, the 10 most important pathways, including aminoacyl-tRNA biosynthesis, glycine, serine, and threonine metabolism; nitrogen metabolism, citrate cycle [tricarboxylic acid (TCA) cycle], phenylalanine, tyrosine, and tryptophan biosynthesis; valine, leucine, and isoleucine biosynthesis; glyoxylate and dicarboxylate metabolism; alanine, aspartate, and glutamate metabolism; pantothenate and CoA biosynthesis, and cyanoamino acid metabolism, were enriched (Supplementary Table S3).

#### Correlation Analysis Between Changes in Plasma Differential Metabolites and Cardiometabolic Health

**Figure 3:**
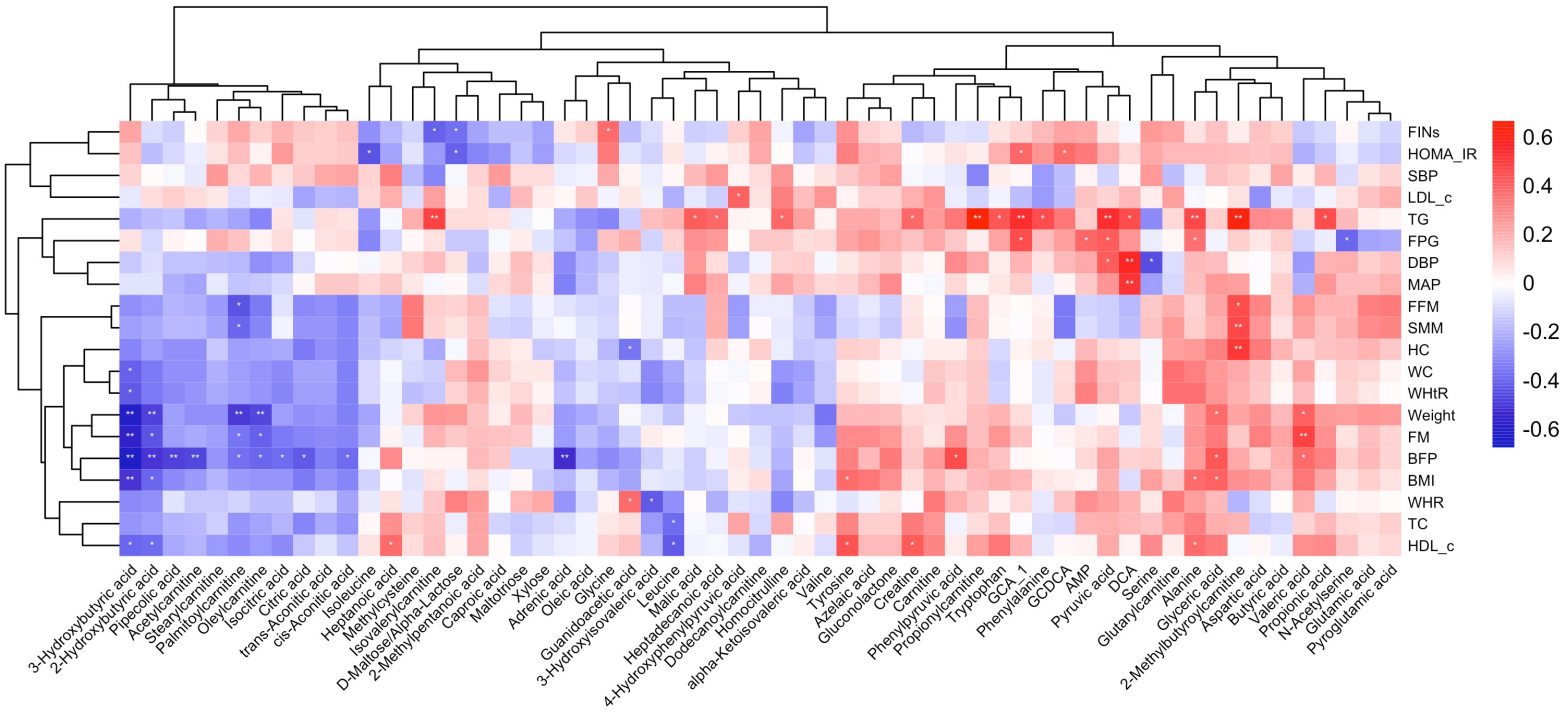
Heat map of the correlations between different metabolites and metabolic improvement. Each square represents the Spearman’s correlation coefficient with the statistical significance threshold set at ^*^p<0.05, ^**^p<0.01. Red and blue colors represent positive and negative correlations, respectively.

To further explore the changes in differential metabolites association with body composition and cardiometabolic health improvement, we conducted a correlation analysis between 59 potential metabolites and cardiometabolic risk factors; the correlation analysis heat map is shown in Figure 3. In our results, a range of different metabolites correlated with the improvements in body composition. The changes in 3-hydroxybutyric acid, isocitric acid, citric acid, trans-aconitic acid, 2-hydroxybutyric acid, pipecolic acid, acetylcarnitine, palmitoyl carnitine, and oleylcarnitine were negatively correlated with the changes in body composition, whereas the changes in valeric acid, glyceric acid, and 2-methylbutyroylcarnitine showed a positive correlation. Correlation analysis of differential metabolites with glucose metabolic outcomes showed that the levels of D-maltose/alpha-lactose were negatively correlated with HOMA-IR and FIN changes. The changes in GCA_1 was positively correlated with the changes in HOMA-IR and FPG, whereas changes in AMP, pyruvic, and alanine were positively correlated with the change in FPG. For the outcomes of lipid metabolism, a positive correlation existed between the changes in TG and a large number of differential metabolites, including GCA_1, tryptophan, alanine, 2-methybutyroycarntine, propionyl carnitine, propionic acid, creatine, isovalerylcarnitine, DCA, pyruvic acid, malic acid, et al. The changes in HDL-c and TC were negatively correlated with leucine. The changes in pyruvic acid, DCA, were positively correlated with the improvement of blood pressure, as observed in our study (Figure 3).

## Discussion

Obesity is a risk factor for metabolic syndrome, and metabolic syndrome in childhood increases the risk of metabolic diseases in adulthood. Metabolites, as biomarkers for the early diagnosis of diseases, play an important role in revealing the early changes in diseases. Changes in plasma metabolites are closely related to metabolic abnormalities in obese children (Newgard et al., 2009; Wahl et al., 2012; Butte et al., 2015). Although extensive research has been carried out to analyze the effects of extreme weight loss programs, no study evaluated the effect of extreme weight loss programs on circulating metabolites and their relationship with cardiometabolic health in children with metabolic syndrome. To our knowledge, this research is the first study to investigate the effects of an extreme weight loss intervention based on exercise combined with diet control on plasma metabolite profile in children with metabolic syndrome. In our study, the dietary control was designed in reference to RMR. Thus, exercise may play an important role in improving body composition. Our results showed that the body composition and cardiometabolic risk factors improved, and 59 metabolites, including amino acids, fatty acids, carnitine, carbohydrates, and organic acids, were changed after intervention.

### Effects of Extreme Weight Loss Intervention on Body Composition and Cardiometabolic Health

In our study, the body composition outcomes, including body weight, BMI, BFP, and FM, decreased after the intervention, with Cohen’s *d* greater than 0.8, indicating that the extreme weight loss intervention can cause substantial improvement in body composition. Although moderate-intensity exercise without diet control can reduce the weight of obese children, the effect size is small. Mendelson et al. (2015) observed that a 12-week exercise program consisting of 60–80% VO_2peak_ exercise intensity, 60–120min train session, and three times/week frequency had a limited effect on weight loss and lipid metabolism. In our study, a large improvement in the body composition might have played an important role in weight maintenance. A great weight loss at the beginning of treatment has been identified as a predictor of long-term weight loss success and maintenance (Nackers et al., 2010). In addition to the improved body composition, our intervention showed an enhancement in glucose metabolism, lipid metabolism, and blood pressure. This finding is consistent with that of Kerns et al. (2017), who reported that diet restriction and vigorous physical activity intervention in the Biggest Loser Competition can achieve the rapid loss of massive weight and that the maintenance of weight loss depends on physical activity changes rather than dietary intake changes during the 6-year follow-up. Liu et al. (2015) discovered that after 4weeks of aerobic exercise combined with dietary intervention, glucose metabolism parameters, such as FINs, HOMA-IR, and HOMA-β, decreased in obese children, indicating that insulin sensitivity in obese children was effectively improved. Given these results, the extreme weight loss intervention based on exercise combined with dietary control may be a more effective intervention for improving body composition and metabolic health in children with metabolic syndrome.

### Effects of Extreme Weight Loss Intervention on Amino Acid Metabolism and Potential Metabolite Pathway

Obesity can cause changes in amino acid metabolism, which is closely related to glucose and lipid metabolism in individuals with obesity (Newgard, 2012; McCormack et al., 2013; Lee et al., 2015). Perng et al. (2014) observed that the concentration of plasma BCAAs in children with obesity was higher than that in children with normal weight, and each unit increase in BCAAs resulted in a corresponding 6% increase in HOMA-IR. BCAAs and AAAs (phenylalanine and tyrosine) together constitute PC6, which is closely related to the occurrence of insulin resistance (Butte et al., 2015). In this study, we observed that BCAAs, including leucine and valine, and AAAs, including tyrosine, phenylalanine, and tryptophan, decreased after the intervention. Given their critical role in insulin resistance, the changes in BCAAs and AAAs may play an important role in maintaining metabolic health. Metabolic pathway analysis further supported this hypothesis and revealed that aminoacyl-tRNA biosynthesis, nitrogen metabolism, phenylalanine, tyrosine, and tryptophan biosynthesis; and valine-leucine and isoleucine biosynthesis were enriched as the most critical metabolic pathways, and BCAAs and AAAs are important metabolites of these metabolic pathways. This finding is consistent with that of Chen et al. (2015), who observed that weight loss was associated with the decrease in BCAAs (leucine and isoleucine) and AAAs (tyrosine and phenylalanine); the decrease in tyrosine and phenylalanine was associated with the improvement of insulin resistance, and this effect was independent of weight change. In our study, we also observed that changes in amino acid metabolites were strongly associated with improved body composition and cardiometabolic risk factors. Aspartic acid and alanine are amino acid metabolites in the aminoacyl-tRNA biosynthesis and alanine, aspartate, and glutamate metabolism, which decreased after the intervention; the change in alanine was positively correlated with the changes in TG, non-HDL-c, TC, BMI, and FPG. This result was consistent with that of Brennan et al. (2018a), who reported that the change in alanine is positively associated with the change in BMI after a regular exercise. A low plasma glycine level is closely correlated with the occurrence of obesity, type 2 diabetes, and non-alcoholic fatty liver disease (Guasch-Ferré et al., 2016; Gaggini et al., 2018), and the level of plasma glycine is positively correlated with insulin sensitivity (Takashina et al., 2016); precursors of glycine, such as trimethylglycine and dimethylglycine, can reduce the risk of diabetes (Svingen et al., 2016). Our results showed that the levels of glycine and serine increased after the intervention, and the change in glycine is positively correlated with HOMA-IR and FIN improvement, suggesting that elevated plasma glycine levels may play an important role in insulin resistance improvement. This finding was also reported by Palmnäs et al. (2018), who observed that low serum serine and glycine levels in adult males were associated with increased body fat and risk of metabolic syndrome, whereas an increased physical activity energy expenditure was positively correlated with increased serum serine and glycine levels.

### Effects of Extreme Weight Loss Intervention on Fatty Acid Metabolism

Fatty acid composition can provide valuable information on the diagnosis of diseases and can be used as a biomarker to evaluate disease status (Bogie et al., 2020; Schjødt et al., 2020; Huang et al., 2021). Based on the carbon chain length, fatty acids can be divided into short-chain fatty acids (SCFAs), medium-chain fatty acids, and long-chain fatty acids. SCFAs are vital energy and signaling molecules produced by microbial fermentation (Koh et al., 2016). SCFAs are increasingly being accepted to play an important role in human health. Riva et al. (2017) observed that childhood obesity is associated with altered gut microbiota, and that the levels of SCFAs produced by gut bacteria are higher than those of normal-weight children. The results of Goffredo et al. are consistent with those of Riva’s; the plasma concentrations of SCFAs, such as acetate, propionate, and butyrate, were positively correlated with the degree of adiposity in children independent of age, gender, and ethnicity (Goffredo et al., 2016). These results suggest that the increased plasma SCFA concentrations were associated with obesity; several research showed that the association between SCFAs and obesity may be bidirectional, and obesity may have an effect on SCFA metabolism (Sowah et al., 2019). In this study, we observed that five SCFAs, including 3-hydroxyisovaleric acid, propionic acid, valeric acid, 2-methylvaleric acid, and caproic acid, were reduced after the intervention. The changes in propionic acid, valeric acid, and caproic acid were associated with the improvements in TG, weight and FM, and SBP, respectively. The decrease in SCFA after weight loss was similar to that of previous systematic review. Sowah et al. conducted a systematic review and discovered that the decreases in SCFA concentrations may accompany the weight loss induced by bariatric surgery or dietary restriction among overweight and obese adults (Sowah et al., 2019). The decreased in SCFA concentrations in our study may be related to the diet control, because SCFAs are the major products of the anaerobic fermentation of primarily nondigestible carbohydrates by the gut microbiome. In addition, a relatively limited number of studies reported the effects of exercise on intestinal flora and SCFAs. The role of exercise in the reduction of SCFA concentration still needs further study. Furthermore, our results revealed that azelaic acid, heptanoic acid, and heptadecanoic acid decreased, whereas 2-hydroxy-3-methyl butyric acid, adrenic acid, and oleic acid increased following the intervention, indicating that exercise plus diet improves the fatty acid metabolism. This finding is consistent with that of Guo et al., who observed that serum total fatty acids, unsaturated fatty acids, monounsaturated fatty acids, polyunsaturated fatty acids, and N-6 polyunsaturated fatty acids reduced after 16weeks of exercise plus diet (Guo et al., 2014).

### Effects of Extreme Weight Loss Intervention on Carnitine Metabolism

Acylcarnitine is a product of the incomplete oxidation of fatty acids. High levels of BCAAs interfere with the oxidation of fatty acids in muscles, leading to the accumulation of various acylcarnitines and insulin resistance (Olisja et al., 2015; White et al., 2016). Wahl et al. (2012) observed that the C12:1 and C16:1 acylcarnitine levels in children with obesity were higher than those in children with normal weight. Perng et al. (2014) also discovered a positive correlation between C3 and C5 acylcarnitine and insulin resistance in children with obesity. Our results showed that carnitine, propionyl carnitine, 2-methylbutyroylcarnitine, isovalerylcarnitine, glutaryl carnitine, and dodecanoyl carnitine decreased, whereas acetylcarnitine, palmitoyl carnitine, oleylcarnitine, and stearylcarnitine increased following the intervention. The changes in propionylcarnitine and isovalerylcarnitine were associated with the changes in TG, whereas those in acetylcarnitine, palmitoyl carnitine, and oleylcarnitine were associated with body composition improvement. This condition may be associated with a reduction in body weight and concentrations of energy fatty acids after the intervention, whereas the adaptive decrease in carnitine content may be the result of improved lipid metabolism.

### Effects of Extreme Weight Loss Intervention on Carbohydrate and Organic Acid Metabolism

Carbohydrate metabolism showed a similar trend to fatty acid and carnitine metabolisms, which decreased following the intervention. Given that fatty acids and carbohydrate compounds are energy substances, the decrease in fatty acid and carbohydrate metabolites after the intervention suggests an increase in the energy metabolic pathway. The TCA cycle is a key link for the metabolism of carbohydrates, fatty acids, and amino acids. Previous studies have revealed damage to the TCA cycle in individuals with obesity and diabetes, which is manifested by the decrease in key metabolites, such as citric acid, α-ketoglutarate, malic acid, and oxaloacetic acid, in the TCA cycle pathway; the damage to the TCA cycle is closely related to insulin resistance (Schrauwen and Hesselink, 2008; Martins et al., 2018). Citric acid, isocitrate, cis-aconic acid, and malic acid are important metabolites in the TCA cycle; increases in these organic acids indicate an increase in the TCA cycle pathway. Similar to the results of this study, Menshikova et al. (2007) revealed that moderate-intensity aerobic exercise increased citrate synthase activity by 29% in adults with obesity. Brennan et al. (2018b) observed that the increase in TCA cycle metabolites was associated with the decreased visceral fat following 6months of exercise. Furthermore, our results showed that 2-hydroxybutyric, cis-aconitic acid, citric acid, and isocitric acid were associated with body composition improvement, whereas malic acid was associated with TG reduction. Altogether, these results suggest that the TCA cycle and organic acid metabolism may play an important role following intervention and are correlated with obesity and its metabolic complications.

Strength and limitation: In accordance with the Convention on the Rights of the Child, we informed the children about the research, and in the implementation of our intervention program, we respected the children’s appeals and rights and encouraged them to complete the intervention program. Compared with previous studies, the advantage of our study is that it can achieve a great weight loss effect in a short period and improve metabolic health. Our study may provide references for the development of effective intervention strategies for children with metabolic syndrome. In our study, we observed that the changes in plasma metabolites were closely associated with the improvement in body composition and cardiometabolic health, which may provide a new research perspective for further exploration of metabolic mechanisms. This study presents several limitations. First, the absence of a control group for diet intervention alone restricted the interpretation of the effects of exercise on metabolic responses. Second, given the lack of sample size, the metabolomic findings could not be further validated in our study. We will continue to carry out relevant verification work in the future. Third, although the body composition and cardiometabolic health improved in our study, the long-term outcomes of the intervention may be different, and further studies with a large sample size and long intervention duration should be carried out.

## Conclusion

In conclusion, the most evident finding of this study is that extreme weight loss intervention can effectively improve body composition and cardiometabolic health in children with metabolic syndrome in a short intervention period. The metabolomic data provided a comprehensive view of circulating metabolite changes after exercise combined with diet control; these changes included amino acid, fatty acid, carnitine, and organic acid metabolism. The changes in plasma metabolites are closely associated with body and cardiometabolic health improvement, which provides a new perspective for the study of the mechanism of exercise combined with diet control to promote cardiometabolic health. Additional research is necessary to further validate the result and determine the key metabolism pathway related to cardiometabolic health improvement in children with metabolic syndrome.

## Data Availability Statement

The original contributions presented in the study are included in the article/Supplementary Material, further inquiries can be directed to the corresponding authors.

## Ethics Statement

The studies involving human participants were reviewed and approved by the Ethical Committee of the Guangzhou Sport University. Written informed consent to participate in this study was provided by the participants’ legal guardian/next of kin.

## Author Contributions

JLiu and LZ conceived and carried out experiments. JLiu and JLia conceived experiments and analyzed data. XL carried out experiments. All authors were involved in writing the paper and had final approval of the submitted and published versions.

## Funding

This work was supported by the National Social Science Fund of China (No. 18BTY075), the Project Supported by Guangdong Province Universities and Colleges Pearl River Scholar Funded Scheme (2019), the Key Project of the National Social Science Foundation of China (No. 19ZDA352) and Guangdong Provincial Key Laboratory of Physical Activity and Health Promotion (No. 2021B1212040014).

## Conflict of Interest

The authors declare that the research was conducted in the absence of any commercial or financial relationships that could be construed as a potential conflict of interest.

## Publisher’s Note

All claims expressed in this article are solely those of the authors and do not necessarily represent those of their affiliated organizations, or those of the publisher, the editors and the reviewers. Any product that may be evaluated in this article, or claim that may be made by its manufacturer, is not guaranteed or endorsed by the publisher.

## Acknowledgments

We gratefully acknowledge the valuable contributions of THE BIGGEST LOSER in data collection.

## Footnotes

^1^ http://cran.r-project.org/
