# Supplementary material for: Effects of Extreme Weight Loss on Cardiometabolic Health in Children With Metabolic Syndrome: A Metabolomic Study
Source: Front Physiol. 2021 Sep 24;12:731762. doi: 10.3389/fphys.2021.731762 (PMC8498573; doi:10.3389/fphys.2021.731762)
Supplement: Supplementary file 1 [file Table_1.DOCX]

Table S1 A one-week schedule of training programs

|  | Monday | Tuesday | Wednesday | Thursday | Friday | Saturday | Sunday |
| --- | --- | --- | --- | --- | --- | --- | --- |
| 08:00 - 08:30 | breakfast | breakfast | breakfast | breakfast | breakfast | breakfast | breakfast |
| 08:30-09:30 | Tidying up the dorm | Tidying up the dorm | Tidying up the dorm | Tidying up the dorm | Tidying up the dorm | Tidying up the dorm | Tidying up the dorm |
| 09:30-11:30 | Exercise | Exercise | Exercise | Exercise | Exercise | Exercise | Exercise |
| 11:30-12:00 | Lunch | Lunch | Lunch | Lunch | Lunch | Lunch | Lunch |
| 12:00-15:30 | Resting | Resting | Resting | Resting | Resting | Resting | Resting |
| 15:30-17:30 | Exercise | Exercise | Exercise | Exercise | Exercise | Exercise | Exercise |
| 17:30-18:00 | Dinner | Dinner | Dinner | Dinner | Dinner | Dinner | Dinner |
| 18:00-21:30 | homework | homework | homework | homework | homework | homework | homework |

Exercise item: outdoor hiking, fast walking, jogging, sports games, aerobic exercises, recreational ball games, boxing, high-intensity interval training.
