# Supplementary figures and images for: Effects of Extreme Weight Loss on Cardiometabolic Health in Children With Metabolic Syndrome: A Metabolomic Study
Source: Front Physiol. 2021 Sep 24;12:731762. doi: 10.3389/fphys.2021.731762 (PMC8498573; doi:10.3389/fphys.2021.731762)

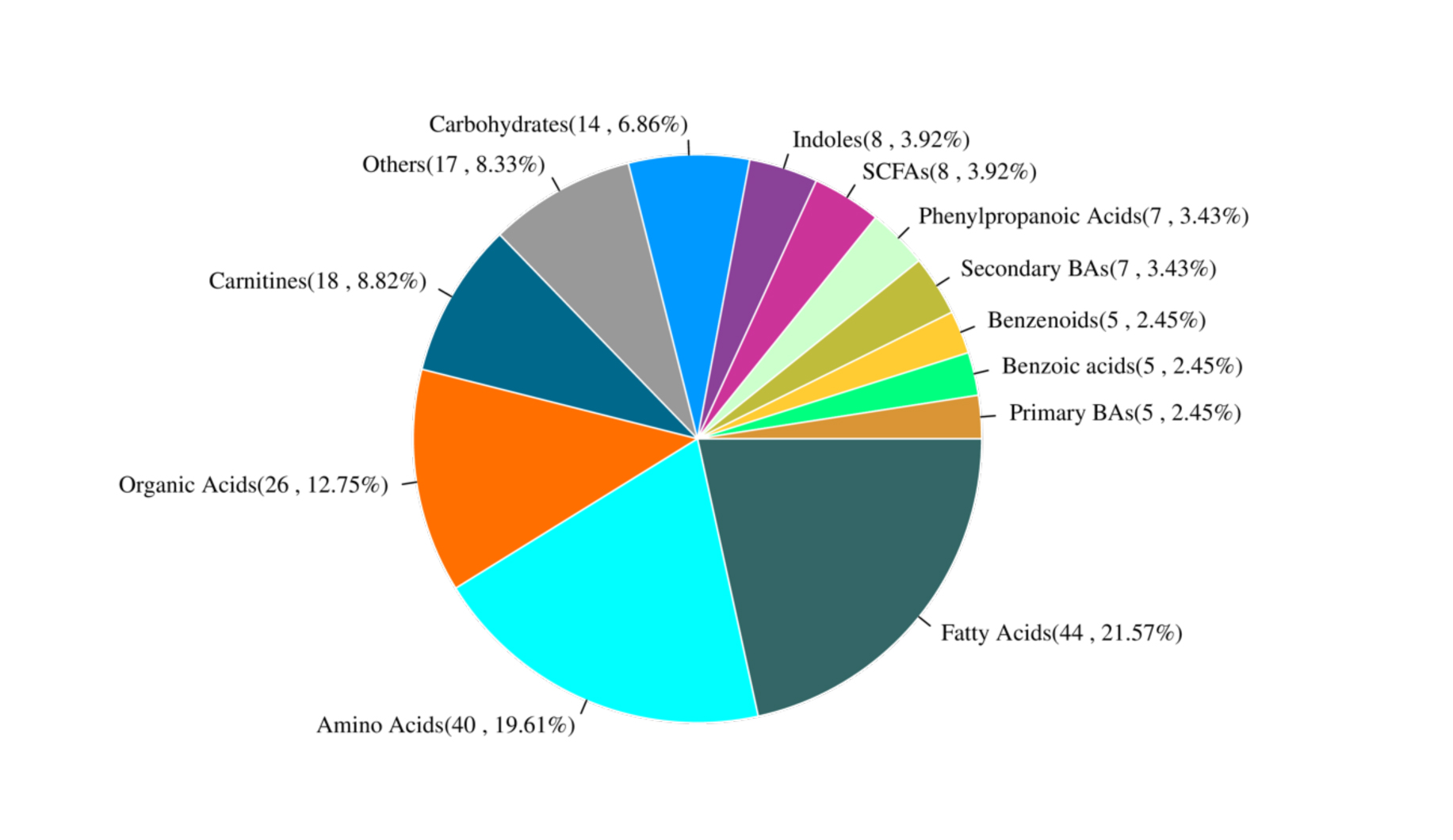

Supplement: Supplementary file 4 [file Image_1.JPEG]
